# Condition-dependent growth and physiological responses of interspecific *Ulva* hybrids under combined temperature-related stresses

**DOI:** 10.3389/fpls.2026.1946853

**Published:** 2026-09-07

**Authors:** Zhengzhao Xu, Xin Wang, Jin Zhao, Peng Jiang

**Affiliations:** 1 Laboratory of Experimental Marine Biology, Institute of Oceanology, Chinese Academy of Sciences, Qingdao, China; 2 Laboratory for Marine Biology and Biotechnology, Qingdao Marine Science and Technology Center, Qingdao, China; 3 University of Chinese Academy of Sciences, Beijing, China; 4 Rizhao Polytechnic University, Rizhao, China

**Keywords:** combined stress, environmental adaptation, interspecific hybridization, physiological performance, *Ulva linza*, *Ulva prolifera*

## Abstract

Interspecific hybridization can generate novel physiological variation, but its consequences for stress tolerance in marine green macroalgae remain poorly understood. In this study, we investigated the growth and photosynthetic pigment responses of parental *Ulva prolifera*, parental *Ulva linza*, and their hybrid offspring under two types of combined environmental stress: temperature × salinity and temperature × desiccation. Parental strains, an F_1_ hybrid sporophyte, and two F_1_ meiotically derived gametophyte lines were exposed to factorial stress treatments for 12 days. Specific growth rate, final wet biomass, and chlorophyll a, chlorophyll b, and carotenoid contents were measured to evaluate growth performance and pigment maintenance. Hybrid offspring did not consistently outperform the parental strains across all treatments, indicating that hybridization did not produce a universal advantage. However, condition-dependent better performance was observed under specific stress combinations. Under temperature × salinity stress, the F_1_ hybrid sporophyte maintained relatively high biomass under some specific temperature–salinity combinations, whereas the F_1_-derived gametophyte lines retained higher biomass or pigment levels under certain high-temperature or high-salinity treatments. Under temperature × desiccation stress, the F_1_ hybrid sporophyte showed relatively stable pigment maintenance across temperature and water treatments, while the F_1_-derived gametophyte lines exhibited stronger strain-specific variation. These results suggest that interspecific hybridization between *U. prolifera* and *U. linza* can broaden physiological variation under fluctuating environmental conditions, but hybrid performance is strongly dependent on stress combinations, life-history stage, and genotype. This study provides new insight into the potential role of hybridization in shaping adaptive responses of green tide-associated *Ulva* taxa in dynamic coastal habitats.

## 1 Introduction

The genus *Ulva* comprises green macroalgae widely distributed in intertidal and nearshore coastal habitats (Xie et al., 2020b). Species such as *Ulva prolifera* are known to form massive green tides that significantly impact coastal ecosystems and local economies (Duong et al., 2025). Large-scale green tides have frequently occurred in the Yellow Sea, with *U. prolifera* typically dominating bloom events, highlighting the ecological and environmental significance of this species. Understanding the mechanisms that generate physiological variation in green tide-associated *Ulva* populations is therefore important for evaluating their adaptive potential under changing coastal environments.

Hybridization is a widespread evolutionary process that generates novel genetic and phenotypic variation through the recombination of divergent genomes, potentially enhancing adaptation to complex environments (Rieseberg et al., 1999). Such variation may manifest as heterosis, transgressive segregation, or the expression of previously cryptic allelic combinations, enabling hybrids to outperform their parental taxa (Goulet et al., 2017). However, hybrid performance is not universally superior across all environments; it is often trait-dependent and environment-specific (Arnold and Martin, 2010; Yu et al., 2020; Wang et al., 2024). Empirical studies indicate that hybrid offspring may exhibit enhanced growth and stress tolerance under some environmental conditions but reduced performance under others (Hatfield and Schluter, 1999; Lippman and Zamir, 2007). In marine macroalgae, interspecific hybridization has been shown to generate novel or enhanced phenotypes relative to parental taxa. For example, hybrids between closely related *Ulva* species differ from their parental strains in growth characteristics, somatic hybrids between *Ulva reticulata* and *Monostroma oxyspermum* exhibit altered morphology and improved agronomic traits, and kelp hybrids between *Laminaria digitata* and *L. pallida* show hybrid vigor for thermal tolerance (Gupta et al., 2015; Martins et al., 2019; Xie et al., 2020a). In coastal habitats, the expression of hybrid advantage is likely to depend on key environmental stressors experienced by macroalgae.

Temperature, salinity, and desiccation were selected in this study because they represent major environmental gradients experienced by *Ulva* species in intertidal, nearshore, and green tide-associated habitats. Temperature is a key environmental factor influencing the growth, photosynthetic performance, and metabolic rate of macroalgae (Raven and Geider, 1988; Cui et al., 2015; Yamaha et al., 2025). Salinity fluctuations directly affect algal osmotic regulation, ionic balance, and cellular water status (Kirst, 1990), while desiccation is a frequent stress encountered by intertidal and floating macroalgae in natural habitats (Davison and Pearson, 1996). Previous studies have shown that *Ulva* species exhibit a certain degree of tolerance to temperature, salinity, and desiccation stress; however, responses vary among species, genotypes, and life-history stages (Cui et al., 2015; Li et al., 2016; Steinhagen et al., 2022). Importantly, environmental stresses rarely occur in isolation in natural ecosystems but often act in combination, so single-factor experiments may not fully reveal the true adaptive capacity of these macroalgae (Lange and Marshall, 2017; Boyd et al., 2018). In Yellow Sea green tide source areas, *Ulva* populations may experience concurrent temperature and salinity fluctuations, while intertidal or stranded thalli may experience temperature variation together with periodic desiccation. Therefore, temperature × salinity and temperature × desiccation treatments were chosen to represent two ecologically relevant combined-stress scenarios and to test whether hybrid offspring differ from their parental species in growth and photosynthetic pigment responses.

Previous phylogeographic and culture-based studies have shown that *U. prolifera* and *U. linza* are closely related freshwater, brackish, and marine *Ulva* lineages, and that reproductive compatibility can occur among some of these taxa under laboratory conditions, laboratory crossing experiments have demonstrated that *U. prolifera* and *U. linza* can show partial gamete compatibility and produce interspecific hybrid offspring (Shimada et al., 2008; Hiraoka et al., 2011). Although most evidence for *Ulva* hybridization has come from laboratory crossing experiments, population-level studies suggest that natural hybridization may also occur, Cui detected rare naturally occurring interspecific hybrids among foliose *Ulva* species, indicating that gene flow between closely related *Ulva* taxa is possible in the field (Cui et al., 2018). This is important because even rare hybridization events may introduce novel genetic combinations into local populations, especially in coastal habitats where closely related species co-occur. In coastal regions of China, including Yellow Sea green tide source areas, *U. prolifera* and *U. linza* may overlap spatially and temporally. *U. prolifera* is generally regarded as the dominant species during Yellow Sea green tide events, whereas *U. linza* often occurs in early-stage or source-area *Ulva* communities. Previous studies have shown that large numbers of microscopic propagules are released during green tide development, and active sexual reproduction occurs in Yellow Sea green tide populations (Liu et al., 2022; Zhao et al., 2022a). Although direct field evidence remains insufficient, such ecological overlap and reproductive activity suggest that hybridization may contribute to physiological variation and adaptive potential in green tide-associated *Ulva* populations.

Despite previous studies on hybrid compatibility and environmental responses of closely related *Ulva* species, several knowledge gaps remain. Most interspecific hybridization studies have focused on gamete fusion, zygote formation, or early development of hybrid sporophytes, with limited attention to the physiological performance of hybrids in subsequent life-history stages (Hiraoka et al., 2011, Hiraoka et al., 2017; Xie et al., 2020a). *Ulva* species have an isomorphic alternation of generations, in which diploid sporophytes and haploid gametophytes are morphologically similar but differ in ploidy level and reproductive origin. However, systematic comparisons between hybrid sporophytes and their meiotically derived gametophytes under environmental stress are lacking. Existing physiological studies on *Ulva* generally examine a single species or single environmental factor, and comparative analyses of parental species and hybrids under combined temperature × salinity or temperature × desiccation stresses remain scarce (Gao et al., 2016). Therefore, whether interspecific hybridization can modify offspring growth and photosynthetic performance under complex environmental conditions remains unresolved.

To address these gaps, we used *U. prolifera*, *U. linza*, and their F_1_ hybrids to investigate responses to two classes of combined environmental stresses: temperature × salinity and temperature × desiccation. Growth, biomass accumulation, and photosynthetic pigment content were measured for parental species, F_1_ hybrid sporophytes, and F_1_-derived gametophytes. Based on the concept of environment-dependent hybrid advantage (Wang et al., 2024), we tested the following hypotheses: (i) hybrid offspring exhibit condition-specific adaptive advantages under certain combinations of environmental stresses; (ii) hybrid sporophytes and gametophytes differ in physiological responses due to ploidy and life-history stage; and (iii) meiotically derived F_1_ gametophyte lines display strain-specific variation in growth and photosynthetic pigment responses.

## 2 Materials and methods

### 2.1 Algal material

*U. prolifera* was collected from Xiamen, Fujian, China on 27 April 2024, and *U. linza* was collected from the intertidal zone in Qingdao, Shandong, China on 22 May 2023. The species identity of each strain was confirmed by sequencing the nuclear ITS region and the 5S rDNA intergenic spacer (Shimada et al., 2008). After collection, thalli were rinsed with filtered seawater to remove epiphytes and debris, and healthy fragments were selected for culture. Strains were maintained in Von Stosch Enriched (VSE) medium (Ott, 1965) at 16 °C under a 12 h light/12 h dark photoperiod, with a light intensity of approximately 120 μmol photons m^−2^ s^−1^. Prior to experimental treatments, algal fragments were acclimated under these standard conditions for one week to ensure stable growth and physiological status.

Following collection, the ploidy and mating type of the field-derived strains were initially determined. Among the *U. linza* isolates, one diploid individual was induced to release haploid progeny, from which an mt^+^ haploid gametophyte was selected as the *U. linza* parent. An mt^−^ haploid gametophyte of *U. prolifera* was selected as the other parental strain. Gamete release was induced following the method of Hiraoka (Hiraoka et al., 2003). Interspecific hybridization was conducted on 11 October 2024 using gametes released from *U. linza* mt^+^ and *U. prolifera* mt^−^. Compatible gametes were mixed to generate F_1_ hybrid sporophytes, which were subsequently cultured to maturity and induced to release reproductive cells. The mating type, ploidy, and life-history stage of the parental strains and hybrid offspring were determined using molecular markers located within the sex-determining region of *Ulva*. Haploid mt^+^ and mt^−^ gametophytes each carried only the corresponding mating-type-specific marker, whereas diploid F_1_ sporophytes simultaneously carried both mt^+^ and mt^−^ specific markers. The reproductive cells released by mature F_1_ sporophytes were identified as F_1_-derived haploid gametophytes when only one of the two mating-type-specific markers was detected (Xu et al., 2026). Based on these molecular diagnoses, one F_1_ hybrid sporophyte line and two F_1_-derived gametophyte lines were selected for the physiological experiments.

### 2.2 Experimental design

**Figure 1:**
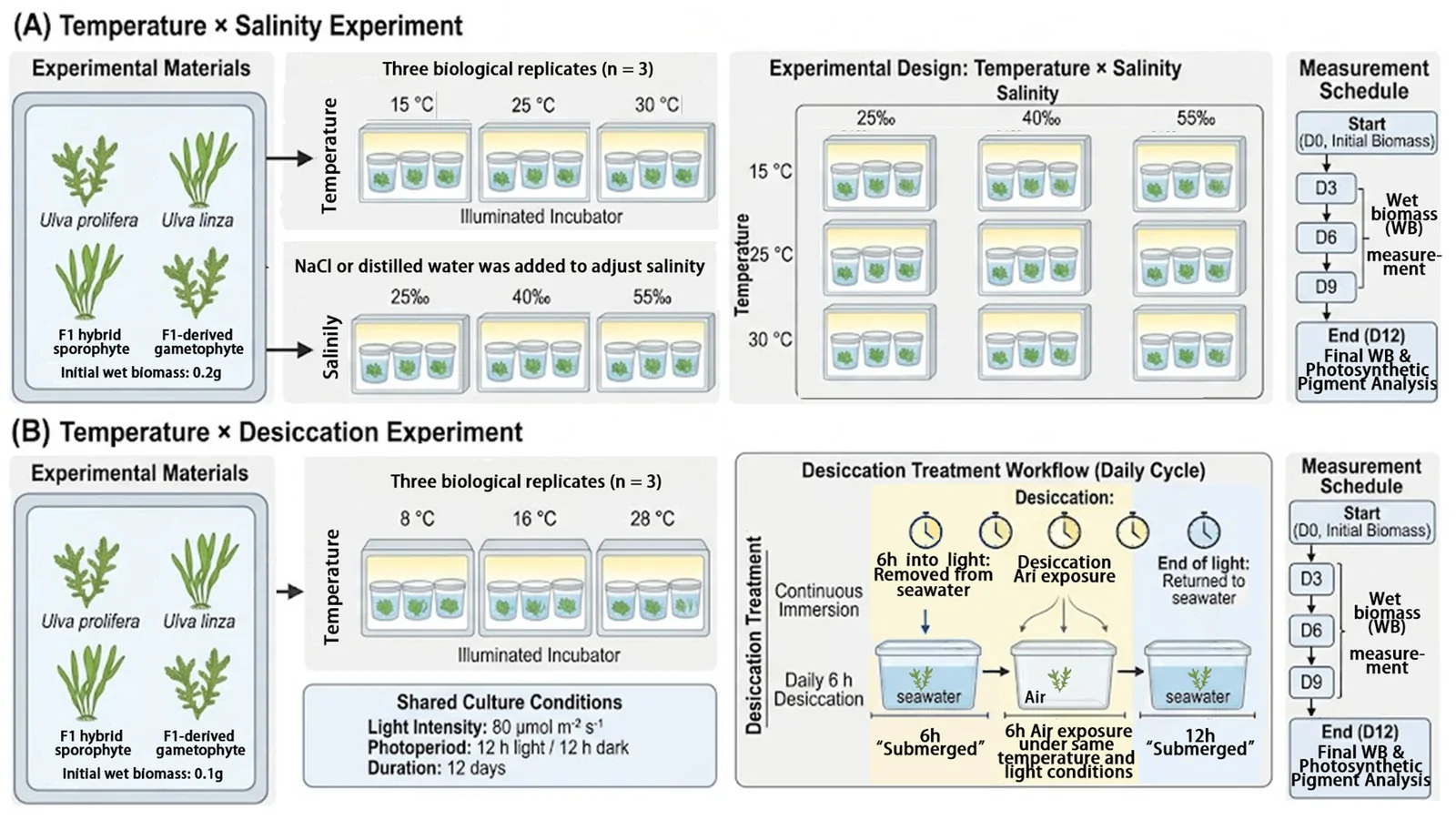
Experimental design and workflow of temperature × salinity and temperature × desiccation stress experiments. Schematic illustration of the experimental design and workflow used to evaluate the physiological responses of parental and hybrid-derived *Ulva* lines under combined environmental stresses. **(A)** Temperature × salinity experiment conducted under three temperature levels (15, 25, and 30 °C) and three salinity levels (25‰, 40‰, and 55‰). **(B)** Temperature × desiccation experiment conducted under three temperature levels (8, 16, and 28 °C) with continuous immersion and daily 6 h desiccation treatments. The experimental materials included the parental gametophytes of *U. prolifera* (UP) and *U. linza* (UL), one F_1_ hybrid sporophyte line (UP×UL1), and two F_1_-derived gametophyte lines (UP×UL2 and UP×UL3).

The physiological experiments began on 7 January 2026 and included two parental gametophyte strains, one F_1_ hybrid sporophyte line, and two F_1_-derived gametophyte lines. To evaluate growth and photosynthetic physiological responses under combined environmental stresses, two factorial experiments were conducted: a temperature × salinity experiment and a temperature × desiccation experiment. The overall experimental workflow and treatment designs are summarized in **Figure 1**. Before the experiments, healthy algal thalli with similar growth status and morphology were selected. Surface water was gently removed with filter paper, and the algal fragments were weighed and placed in 750 mL culture containers for stress treatments. During the experiments, the culture seawater supplemented with VSE medium was renewed every three days. The positions of the culture containers were adjusted regularly within the incubators to ensure uniform light exposure.

#### 2.2.1 Temperature × salinity experiment

The temperature × salinity experiment was conducted using *U. prolifera*, *U. linza*, F_1_ hybrid sporophytes, and F_1_-derived gametophytes as experimental materials. The initial wet biomass (WB) of each algal fragment was 0.20 g, and the experiment lasted for 12 days. A two-factor fully factorial design was used, including three temperature levels and three salinity levels. The temperature treatments were 15, 25, and 30 °C, representing normal culture temperature, moderate heat stress, and high-temperature stress, respectively. Salinity levels of 25‰, 40‰, and 55‰ were selected to represent a gradient from relatively low salinity to moderate and extreme salinity stress. The elevated salinity treatments were designed to simulate transient salinity increases that may occur in intertidal habitats and stranded green-tide algal mats as a result of intense water evaporation. Specifically, 40‰ represented moderate salinity stress, whereas 55‰ represented an extreme hypersaline condition used to evaluate the upper tolerance and physiological responses of the parental and hybrid materials. Salinity was adjusted by adding appropriate amounts of NaCl or freshwater to the culture seawater and was verified using a handheld refractometer. The experiment was conducted in non-aerated culture vessels placed inside an illuminated incubator (GXZ-380B, Ningbo Southeast Instrument Co., Ltd., Ningbo, China). The incubator was used to regulate temperature and photoperiod (12 h light/12 h dark), while light intensity within the culture vessels was measured using a light meter (F941, Fluke Corporation, USA) and maintained at approximately 80 μmol photons m^−2^ s^−1^.

#### 2.2.2 Temperature × desiccation experiment

The temperature × desiccation experiment was also conducted using *U. prolifera*, *U. linza*, F_1_ hybrid sporophytes, and F_1_-derived gametophytes. The initial WB of each algal fragment was 0.10 g, and the experiment lasted for 12 days. A two-factor fully factorial design was applied, with three temperature levels and two desiccation treatments. The temperature treatments were 8, 16, and 28 °C, representing low-temperature stress, normal culture temperature, and heat stress, respectively. The desiccation treatments consisted of daily 6 h desiccation and continuous immersion. For the desiccation treatment, algal fragments were removed from seawater at the sixth hour of the light period each day and exposed to the same temperature and light conditions for 6 h, after which they were transferred back into seawater for continued culture. The non-desiccation treatment remained continuously immersed throughout the experiment. Other culture conditions were the same as those used in the temperature × salinity experiment. Three biological replicates were established for each material under each treatment combination.

### 2.3 Measurements

WB was measured at 3-day intervals throughout the experiment (days 3, 6, 9, and 12). For each measurement, algal fragments were removed from the culture medium, gently blotted with filter paper to remove surface water, and weighed using an electronic balance. Specific growth rate (SGR) was calculated based on changes in wet biomass over each 6-day growth period using the following equation:


$$\text{SGR}=\frac{\ln\left({\text{W}}_{\text{t}}\right)-\ln\left({\text{W}}_{0}\right)}{\text{t}}\times 100.$$

Where W_0_ and W_t_ represent the WB at the beginning and end of each growth period, respectively, and t represents the culture duration in days. SGR was expressed as % d^−1^, in this study, SGR was calculated separately for the early growth period (D0–D6) and the late growth period (D6–D12).

At the end of the experiment, fresh algal material was immediately collected and used for photosynthetic pigment determination. Approximately 0.05 g of fresh thallus was cut into small pieces and ground thoroughly with quartz sand and 5 mL of anhydrous methanol under dark conditions. The extract was kept overnight at 4 °C in the dark and then centrifuged at 8000 rpm for 10 min. The supernatant was collected as the pigment extract, and extraction was repeated when necessary until the algal tissue became nearly colorless. Anhydrous methanol was used as the blank, and absorbance was measured at 665, 652, and 470 nm. Chlorophyll a, chlorophyll b, and carotenoid contents were calculated according to Lichtenthaler’s equations:


$$\text{Chl a}=16.72A_{665}-9.16A_{652},$$

$$\text{Chl b}=34.09A_{652}-15.28A_{665},$$

$$\text{Car}=\frac{1000A_{470}-1.63\text{Chl a}-104.96\text{Chl b}}{221},$$

$$\text{Pigment content}=\frac{C\times V\times D}{WB},$$

where *A* represent the absorbance values of the pigment extract at 665, 652, and 470 nm, respectively. Chl a, Chl b, and Car represent the concentrations of chlorophyll a, chlorophyll b, and carotenoids in the methanol extract, respectively, expressed as μg mL^−1^ (Lichtenthaler, 1987). *C* represents the pigment concentration calculated from the equations above (μg mL^−1^), *V* represents the final extraction volume (mL), *D* represents the dilution factor, and *WB* represents the wet biomass of algal material used for extraction (g). Pigment contents were expressed as μg g^−1^ WB. For each biological replicate, absorbance measurements were performed with three technical replicates, and the mean value was used for subsequent calculation and statistical analysis.

### 2.4 Statistical analysis

For the temperature × salinity experiment, permutational multivariate analysis of variance (PERMANOVA) was performed using the adonis2() function implemented in the R package vegan to evaluate the effects of genotype, temperature, salinity, and their interactions on growth and physiological traits. Similarly, PERMANOVA was conducted for the temperature × desiccation experiment to assess the effects of genotype, temperature, desiccation treatment, and their interactions. Euclidean distance was used for PERMANOVA, and statistical significance was evaluated based on 9999 permutations. Following PERMANOVA, Games–Howell *post hoc* tests were performed for pairwise comparisons among groups. Statistical significance was determined at *P* < 0.05. Here, genotype refers to the two parental strains, one F_1_ hybrid sporophyte line, and two F_1_-derived gametophyte lines. Statistical analyses were performed using IBM SPSS Statistics 19.0 and R (vegan package), and figures were generated using Origin 8.0.

## 3 Results

### 3.1 Growth responses under temperature × salinity stress

**Figure 2:**
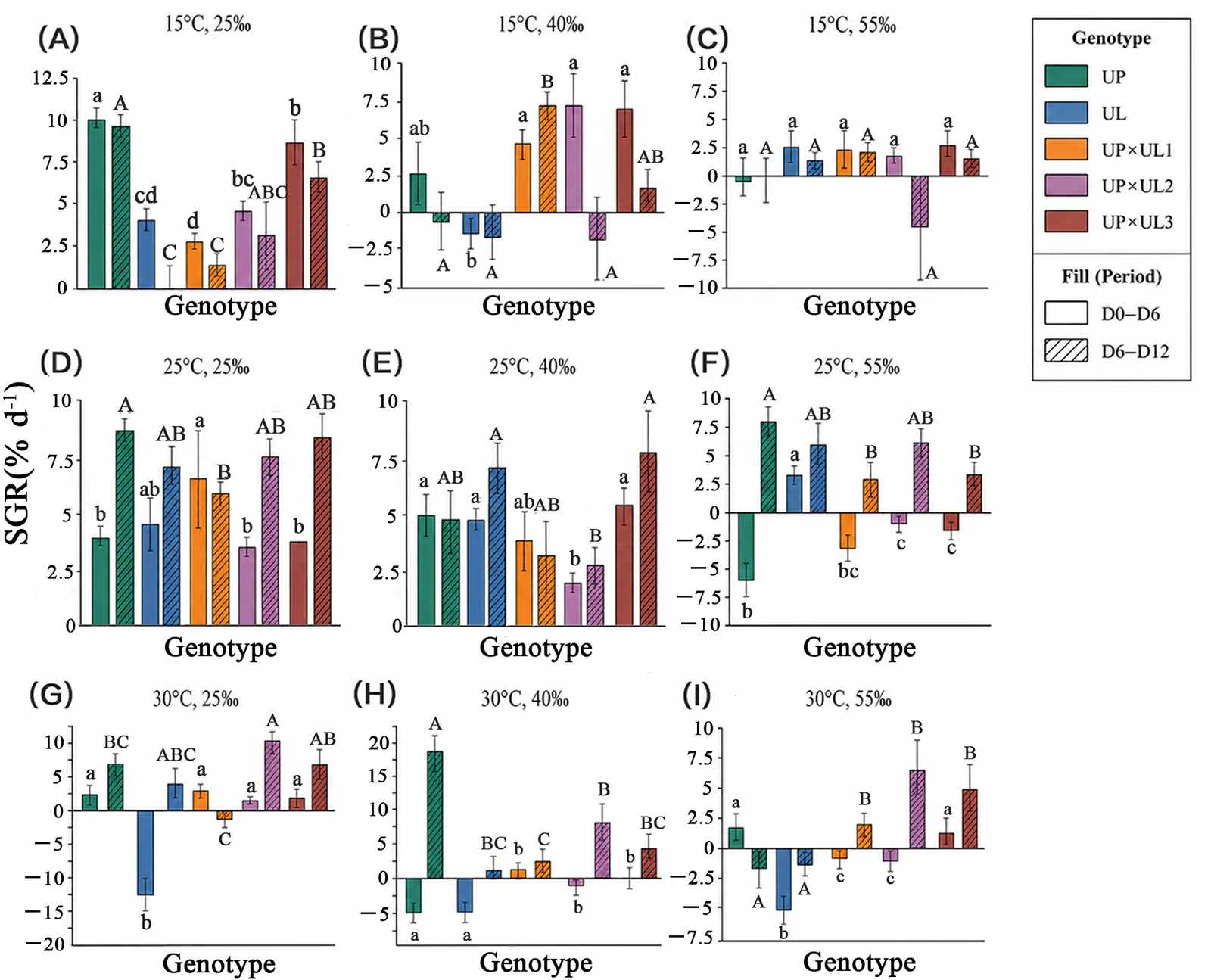
Specific growth rate of parental and hybrid *Ulva* lines under temperature × salinity stress. Specific growth rate (SGR) of *U. prolifera* (UP), *U. linza* (UL), the F_1_ hybrid sporophyte (UP×UL1), and two F_1_-derived gametophyte lines (UP×UL2 and UP×UL3) under different temperature–salinity combinations. Panels **(A–I)** represent combinations of three temperatures (15, 25, and 30 °C) and three salinity levels (25‰, 40‰, and 55‰). SGR was calculated during the early growth period (D0–D6) and late growth period (D6–D12). Data are presented as mean ± SD (n = 3). Different lowercase letters indicate significant differences among genotypes during the early growth period (D0–D6), whereas different uppercase letters indicate significant differences among genotypes during the late growth period (D6–D12) under the same treatment condition, according to Games–Howell multiple comparisons (*P* < 0.05).

Growth responses of parental and hybrid-derived lines varied among different temperature × salinity combinations (**Figure 2**). Significant differences among genotypes were detected under several stress conditions based on Games–Howell multiple comparisons (*P* < 0.05). Three-way PERMANOVA revealed significant effects of genotype, temperature, and salinity on SGR during both growth periods. Significant interactions between genotype and environmental factors further indicated differential growth responses among lines under different temperature and salinity conditions (**Table 1**). At 15 °C × 40‰ salinity, hybrid-derived lines exhibited enhanced growth performance compared with parental strains. The F_1_ hybrid sporophyte UP×UL1 showed sustained growth throughout the experiment, with early- and late-stage SGR values of 4.78 ± 0.97% d^−1^ and 7.15 ± 0.61% d^−1^, respectively, resulting in the highest final biomass (0.410 ± 0.035 g WB). Compared with UL, UP×UL1 exhibited significantly higher SGR during both D0–D6 (*P* = 0.008) and D6–D12 (*P* = 0.040). During D0–D6, both F_1_-derived gametophyte lines UP×UL2 and UP×UL3 exhibited significantly higher SGR than UL (*P* = 0.012 and *P* = 0.005, respectively) (**Figure 2**; **Supplementary Figure 1**).

**Table 1:** Summary of three-way PERMANOVA results for growth and photosynthetic pigment parameters under temperature × salinity stress.

Parameter	G	T	S	G×T	G×S	G×T×S
SGR (D0–D6)	12.253^**^	69.572^**^	27.752^**^	12.299^**^	4.826^**^	5.708^**^
SGR (D6–D12)	13.031^**^	53.383^**^	20.064^**^	14.639^**^	5.779^**^	10.310^**^
Chl a	96.430^**^	88.724^**^	14.118^**^	19.811^**^	8.794^**^	9.904^**^
Chl b	36.500^**^	75.85^**^	7.933^**^	9.301^**^	5.701^**^	3.596^**^
Car	105.875^**^	3.990^*^	6.220^**^	18.483^**^	6.911^**^	5.554^**^
Chl a/Chl b	10.279^**^	2.478^ns^	0.466^ns^	5.759^**^	3.793^**^	5.033^**^

Values are pseudo-F statistics obtained from three-way PERMANOVA with 9999 permutations. G, Genotype; T, temperature; S, salinity. ns, *P* ≥ 0.05; *P < 0.05; **P < 0.01.

### 3.2 Photosynthetic pigment responses under temperature × salinity stress

**Figure 3:**
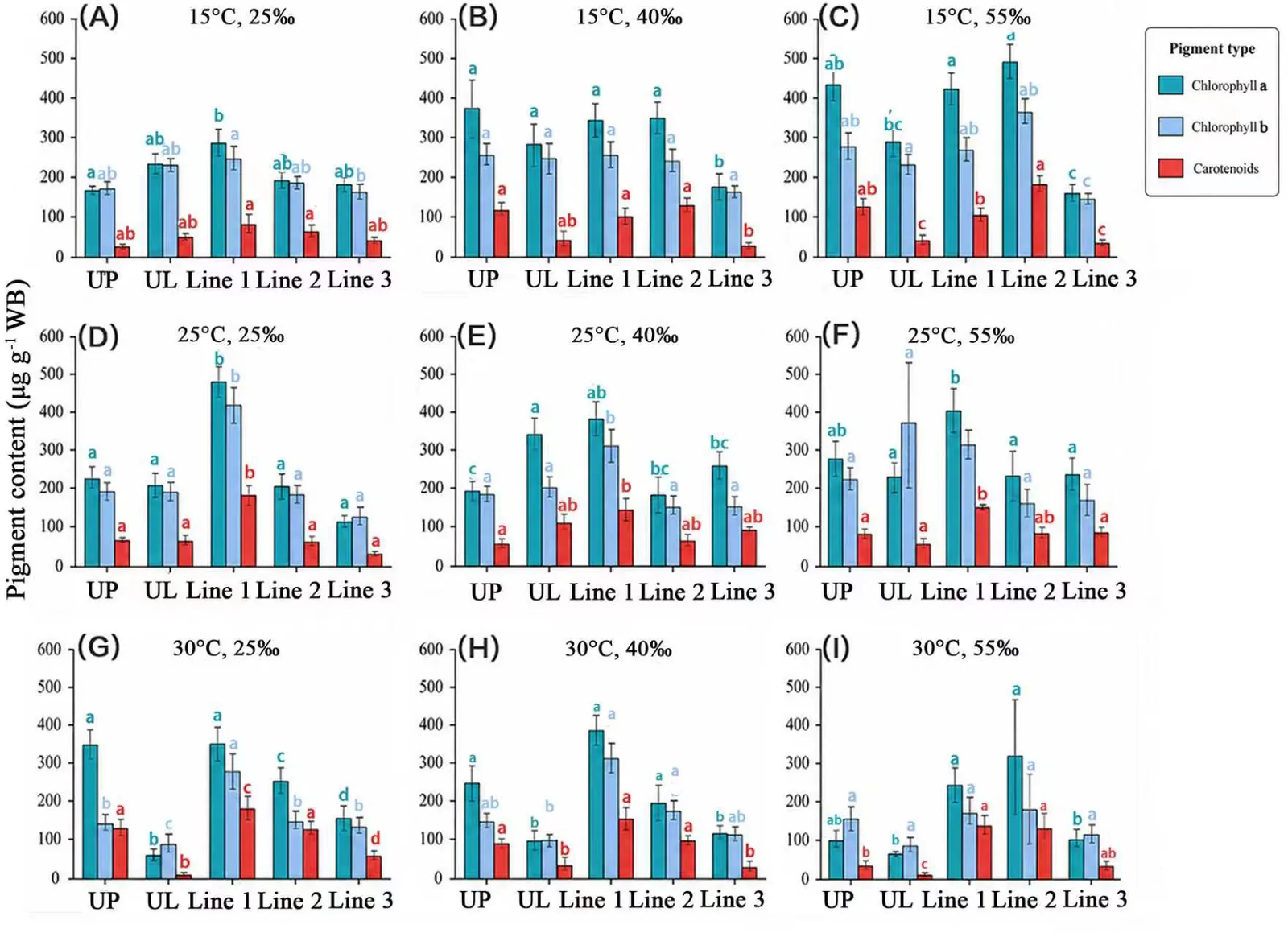
Photosynthetic pigment contents of parental and hybrid *Ulva* lines under temperature × salinity stress. Contents of chlorophyll a (Chl a), chlorophyll b (Chl b), and carotenoids (Car) in *U. prolifera* (UP), *U. linza* (UL), the F_1_ hybrid sporophyte (UP×UL1), and F_1_-derived gametophyte lines (UP×UL2 and UP×UL3) after exposure to different temperature–salinity combinations for 12 days. In the figures, Line 1, Line 2, and Line 3 represent UP×UL1, UP×UL2, and UP×UL3, respectively. Panels **(A–I)** represent different combinations of temperature and salinity treatments. Pigment contents were expressed as μg g^−1^ wet biomass (WB). Data are presented as mean ± SD (n = 3). Different lowercase letters indicate significant differences among genotypes within the same treatment based on Games–Howell multiple comparisons (*P* < 0.05).

Pigment contents of parental and hybrid-derived lines showed distinct responses under different temperature × salinity combinations (**Figure 3**). Significant differences among genotypes were detected for chlorophyll a, chlorophyll b, and carotenoid contents under several conditions based on Games–Howell multiple comparisons (*P* < 0.05). Three-way PERMANOVA revealed significant effects of genotype, temperature, salinity, and their interactions on pigment variation, suggesting that pigment responses varied depending on the combination of genetic background and environmental conditions (**Table 1**). Under moderate temperature conditions (15 °C), hybrid-derived lines showed enhanced pigment maintenance under specific salinity levels. At 15 °C × 55‰ salinity, the F_1_-derived gametophyte line UP×UL2 exhibited the highest chlorophyll a and chlorophyll b contents (497.88 ± 32.34 and 370.74 ± 13.17 μg g^−1^ WB, respectively), which were significantly higher than those of UP and UP×UL3 (*P* < 0.05). Under 15 °C × 40‰ salinity, the F_1_ hybrid sporophyte UP×UL1 and UP×UL2 maintained relatively high chlorophyll contents, whereas UP×UL3 showed lower pigment accumulation. At 25 °C, pigment responses became more genotype-specific. The F_1_ hybrid sporophyte UP×UL1 showed increased chlorophyll accumulation under 25 °C × 25‰ conditions, with chlorophyll a and chlorophyll b contents of 487.28 ± 82.41 and 423.95 ± 67.76 μg g^−1^ WB, respectively, which were significantly higher than those of some parental and hybrid-derived lines (*P* < 0.05). However, under high-temperature conditions (30 °C), pigment responses became more genotype- and salinity-dependent. Under 30 °C × 25‰ salinity, the F_1_-derived gametophyte line UP×UL3 exhibited reduced pigment accumulation, with lower chlorophyll b and carotenoid contents compared with some other lines (*P* < 0.05). Similar reductions in carotenoid content were observed for UP×UL3 under 30 °C × 40‰ and 30 °C × 55‰ conditions, where it showed significantly lower carotenoid levels than the F_1_ hybrid sporophyte UP×UL1 (*P* = 0.020 and *P* = 0.013, respectively). These results indicate that the pigment advantages of hybrid-derived lines were weakened under high-temperature stress and varied among hybrid lineages and salinity conditions. Overall, hybrid-derived lines exhibited temperature- and salinity-dependent pigment responses, with enhanced pigment maintenance under certain stress combinations but without a universal advantage across all environments.

### 3.3 Growth responses under temperature × desiccation stress

**Figure 4:**
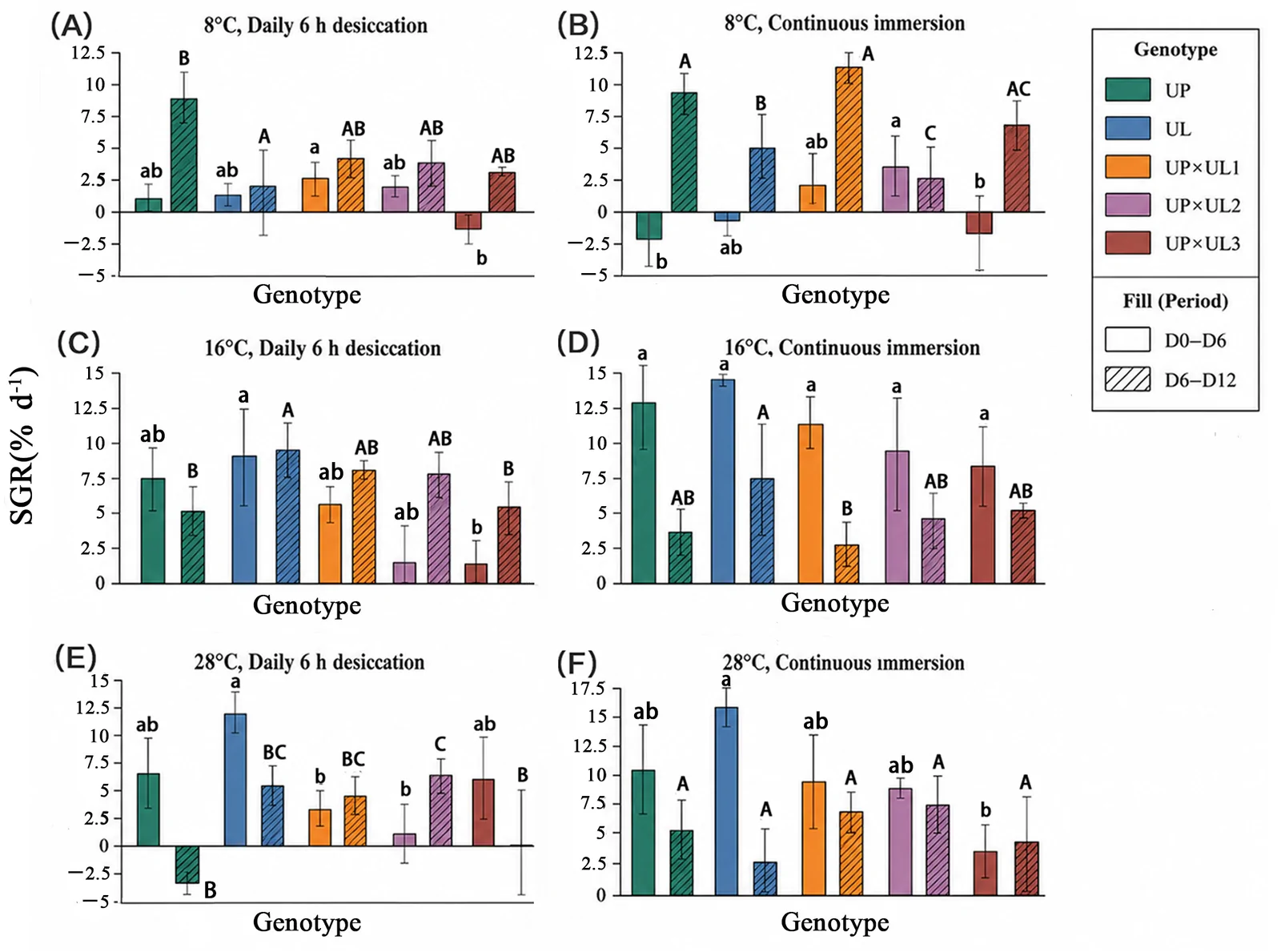
Specific growth rate of parental and hybrid *Ulva* lines under temperature × desiccation stress. Specific growth rate (SGR) of *U. prolifera* (UP), *U. linza* (UL), the F_1_ hybrid sporophyte (UP×UL1), and F_1_-derived gametophyte lines (UP×UL2 and UP×UL3) was evaluated under different temperature and water exposure treatments. Panels **(A–F)** represent different combinations of temperature and water treatments. SGR was calculated during the early growth period (D0–D6) and late growth period (D6–D12). Data are presented as mean ± SD (n = 3). Different lowercase letters indicate significant differences among genotypes during the early growth period (D0–D6), whereas different uppercase letters indicate significant differences among genotypes during the late growth period (D6–D12) under the same treatment condition, according to Games–Howell multiple comparisons (*P* < 0.05).

Growth responses of parental and hybrid-derived lines varied among different temperature × desiccation combinations (**Figure 4**). Significant differences among genotypes were detected under several conditions based on Games–Howell multiple comparisons (*P* < 0.05). Three-way PERMANOVA revealed significant effects of genotype and temperature on SGR during both growth periods, whereas the effect of desiccation treatment varied between the early and late growth periods. Significant interactions between genotype and environmental factors, including genotype × temperature and genotype × temperature × desiccation, indicated that growth responses varied depending on the specific combination of genotype and stress conditions (**Table 2**). At 8 °C under continuous immersion, the F_1_ hybrid sporophyte UP×UL1 maintained a relatively high late-stage SGR of 11.35 ± 0.88% d^−1^ and exhibited significantly higher growth than UL (*P* = 0.029) and the F_1_-derived gametophyte line UP×UL2 (*P* = 0.036), indicating a genotype-specific growth advantage under low-temperature immersion conditions. Under daily desiccation, however, the growth responses of hybrid-derived lines were more variable, with UP×UL3 showing reduced early-stage growth but partial recovery during D6–D12. At 16 °C, parental and hybrid-derived lines generally maintained positive growth under both desiccation and continuous immersion treatments, although their relative performance differed among lines and treatments. Under daily desiccation, UP×UL2 maintained relatively high late-stage SGR, whereas UP×UL3 showed moderate growth recovery after early inhibition. Under continuous immersion, parental lines and hybrid-derived lines showed variable growth patterns, with no consistent superiority of hybrid-derived lines over parental strains (**Figure 4**). Under 28 °C stress, growth responses became more genotype- and treatment-dependent. Under daily desiccation, parental UP exhibited growth inhibition during the late stage (SGR = −3.30 ± 0.94% d^−1^), whereas the hybrid-derived line UP×UL2 maintained positive growth and showed significantly higher late-stage SGR than UP (*P* = 0.027). Under continuous immersion, UL showed relatively high early-stage growth, while hybrid-derived lines displayed intermediate responses without a consistent growth advantage (**Figure 4**; **Supplementary Figure 2**). Overall, hybrid-derived lines did not universally outperform parental strains, but certain hybrid lines maintained enhanced growth under specific temperature × desiccation combinations, indicating environment- and lineage-dependent response.

**Table 2:** Summary of three-way PERMANOVA results for growth and photosynthetic pigment parameters under temperature × desiccation stress.

Parameter	G	T	W	G×T	G×W	G×T×W
SGR (D0–D6)	18.369^**^	101.007^**^	46.568^**^	5.846^**^	2.959^*^	1.529^ns^
SGR (D6–D12)	3.441^*^	12.130^**^	4.226^*^	13.301^**^	5.106^ns^	3.773^**^
Chl a	25.070^**^	26.856^**^	6.795^*^	4.895^**^	3.483^*^	0.526^ns^
Chl b	40.144^**^	47.063^**^	0.003 ^ns^	4.616^**^	4.962^**^	1.398^ns^
Car	31.095^**^	21.211^**^	1.267^ns^	4.401^**^	3.542^*^	1.132^ns^
Chl a/Chl b	19.167^**^	4.661^*^	36.505^**^	10.417^**^	1.844^ns^	5.678^**^

Values are pseudo-F statistics obtained from three-way PERMANOVA with 9999 permutations. G, Genotype; T, temperature; W, water treatment. Water treatment included continuous immersion and daily 6-h desiccation treatments. ns, *P* ≥ 0.05; *P < 0.05; **P < 0.01.

### 3.4 Photosynthetic pigment responses under temperature × desiccation stress

**Figure 5:**
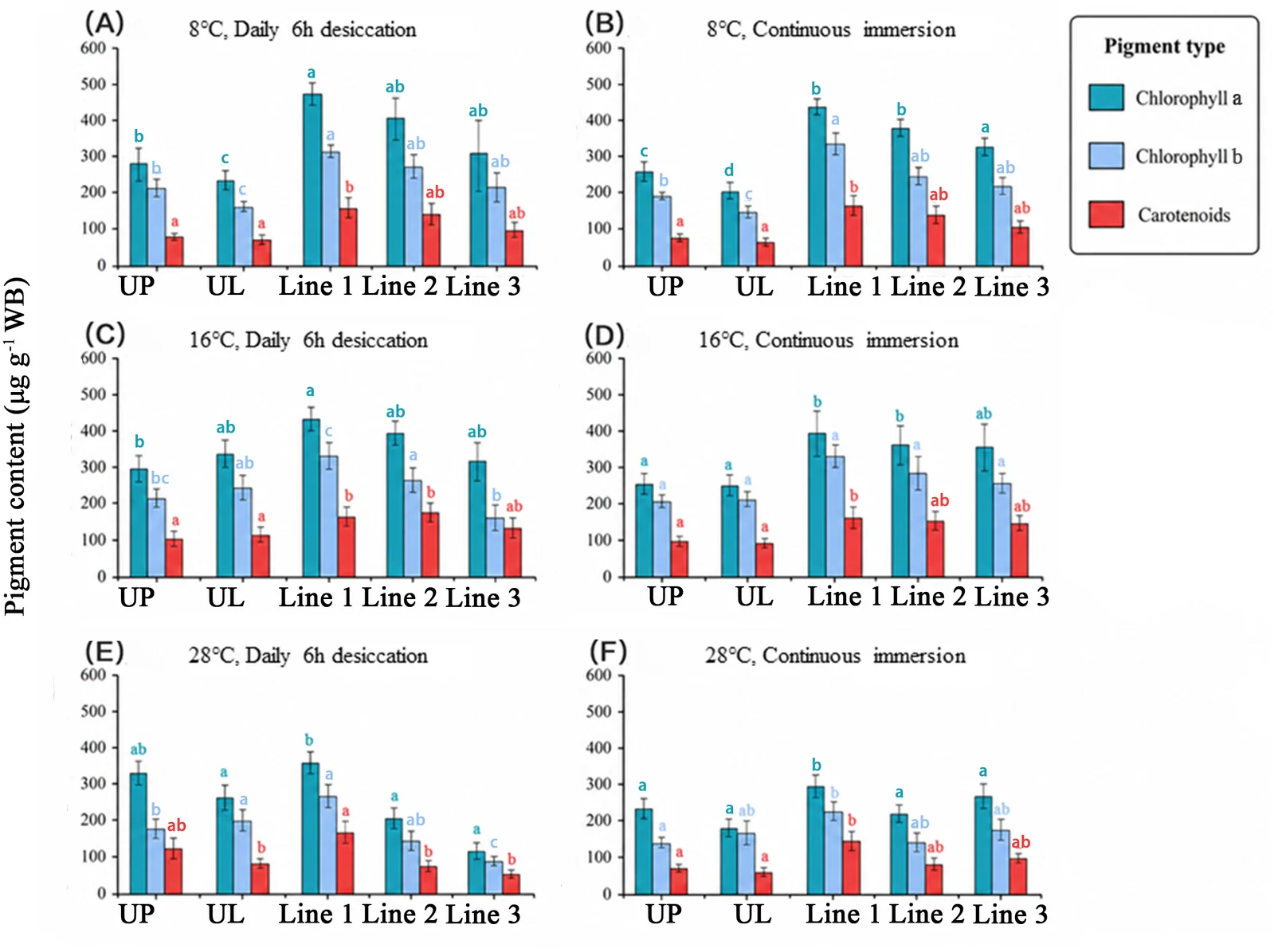
Photosynthetic pigment contents of parental and hybrid *Ulva* under temperature × desiccation stress. Contents of chlorophyll a (Chl a), chlorophyll b (Chl b), and carotenoids (Car) in *U. prolifera* (UP), *U. linza* (UL), the F_1_ hybrid sporophyte (UP×UL1), and F_1_-derived lines (UP×UL2 and UP×UL3) after exposure to different temperature and water availability treatments for 12 days. In the figures, Line 1, Line 2, and Line 3 represent UP×UL1, UP×UL2, and UP×UL3, respectively. Panels **(A–F)** represent different combinations of temperature and water treatments. Pigment contents were expressed as μg g^−1^ wet biomass (WB). Data are presented as mean ± SD (n = 3). Different lowercase letters indicate significant differences among genotypes within the same treatment based on Games–Howell multiple comparisons (*P* < 0.05).

Pigment contents of parental and hybrid-derived lines showed distinct responses under different temperature × desiccation combinations (**Figure 5**). Significant differences among genotypes were detected for chlorophyll a, chlorophyll b, and carotenoid contents under several conditions based on Games–Howell multiple comparisons (*P* < 0.05). Three-way PERMANOVA revealed significant contributions of genotype and temperature to variation in all pigment parameters, whereas the contribution of water treatment varied among pigment traits. Significant genotype × temperature interactions were detected for chlorophyll a, chlorophyll b, carotenoids, and the chlorophyll a/b ratio, indicating that pigment responses varied among lines depending on temperature conditions (**Table 2**).

Under low-temperature daily desiccation conditions (8 °C), the F_1_ hybrid sporophyte UP×UL1 exhibited the highest chlorophyll a and chlorophyll b contents (481.88 ± 26.74 and 311.63 ± 12.06 μg g^−1^ WB, respectively). The chlorophyll a content of UP×UL1 was significantly higher than those of the parental lines UP and UL (*P* = 0.006 and *P* = 0.008, respectively), while its chlorophyll b content was also significantly higher than those of UP and UL (*P* < 0.001 and *P* < 0.001, respectively). UP×UL2 also maintained relatively high pigment levels, whereas UP×UL3 showed intermediate values. Similar patterns were observed under continuous immersion. UP×UL1 exhibited higher chlorophyll a content than both parental lines, with significant differences compared with UP (*P* = 0.011) and UL (*P* < 0.001). UP×UL2 also maintained relatively high chlorophyll a content, showing a significant increase compared with UL (*P* = 0.041), but no significant difference from UP (*P* = 0.128). At 16 °C, UP×UL1 maintained higher chlorophyll and carotenoid contents under both desiccation regimes, while other hybrid-derived lines showed variable responses depending on pigment type and treatment. However, under high-temperature stress (28 °C), the pigment advantages of hybrid-derived lines were reduced, and the gametophyte-derived line UP×UL3 exhibited lower chlorophyll b contents than both parental strains, with significant differences compared with UP (*P* = 0.006) and UL (*P* = 0.003). These results indicate that hybrid-derived lines exhibited temperature- and desiccation-dependent pigment responses, with stronger pigment maintenance under low-to-moderate temperature stress but reduced advantages under high-temperature conditions.

## 4 Discussion

### 4.1 Condition-dependent hybrid advantage under combined stress

We observed distinct growth and photosynthetic pigment responses among *U. prolifera*, *U. linza*, and their F_1_ hybrid offspring under two types of combined stress: temperature × salinity and temperature × desiccation. Notably, hybrid offspring did not consistently outperform the parental strains across all environmental combinations. Instead, higher relative growth rates, biomass accumulation, or photosynthetic pigment contents were observed only under specific temperature, salinity, or desiccation conditions. These findings indicate that the adaptive effects generated by interspecific hybridization between *U. prolifera* and *U. linza* were not expressed as a universal hybrid advantage, but rather as an environment-dependent or condition-dependent hybrid advantage (Wang et al., 2024).

In the temperature × salinity experiment, the F_1_ hybrid sporophyte UP×UL1 showed higher biomass accumulation and relatively stable growth under some moderate-salinity or favorable-temperature combinations, whereas F_1_-derived gametophyte lines displayed lineage-specific responses, with some lines maintaining higher biomass under particular high-temperature or high-salinity treatments. Similarly, in the temperature × desiccation experiment, the F_1_ hybrid sporophyte and specific gametophyte lines maintained relatively higher growth under particular conditions, such as low-temperature immersion or high-temperature desiccation, whereas other hybrid-derived lines showed limited or even reduced performance depending on the treatment. These results suggest that hybrid offspring possess a certain degree of response flexibility under complex environmental conditions. However, under other treatments, differences between hybrids and parental strains were minor, and hybrid superiority was not always observed, indicating that hybridization does not necessarily enhance offspring performance under all environments. In some cases, hybrid offspring may even perform worse than one or both parental species, suggesting that hybridization can also generate maladaptive trait combinations under unfavorable environmental contexts. This further supports the view that hybrid performance is shaped by genotype-by-environment interactions rather than by a fixed superiority of hybrid genotypes (Burke and Arnold, 2001).

This condition-dependent advantage was reflected not only in growth-related traits but also in photosynthetic pigment maintenance. Under some temperature–salinity or temperature–desiccation treatments, hybrid offspring maintained relatively high growth rates, biomass accumulation, and Chl a, Chl b, or carotenoid contents, suggesting better photosynthetic stability and growth maintenance under specific combined-stress conditions. In particular, carotenoids are closely associated with photoprotection and stress buffering, and their relatively high levels in some hybrid offspring may contribute to alleviating photosynthetic pressure caused by high temperature, salinity fluctuation, or desiccation (Xie et al., 2013; Zhao et al., 2022b). However, pigment advantages were not always fully synchronized with growth advantages, indicating that hybrid performance was unlikely to be determined by a single physiological trait, but rather by the combined effects of growth regulation, pigment maintenance, and other stress-response processes.

From an adaptive perspective, these results suggest that interspecific hybridization does not necessarily generate universally superior offspring, but may broaden the range of phenotypic variation across complex environmental gradients (Arnold and Martin, 2010). The fact that different hybrid offspring showed advantages under different treatments implies that novel genetic combinations generated by hybridization may be favored under particular environmental context (Wang et al., 2024). For *Ulva* taxa inhabiting highly fluctuating coastal and intertidal environments, such condition-dependent advantages may help some hybrid progeny maintain growth and physiological activity under specific combined-stress conditions, thereby increasing their potential adaptive space (Zou et al., 2007; Bischof and Rautenberger, 2012; Flores-Molina et al., 2014). This may also have implications for future green tide risk, because hybrid progeny with enhanced tolerance to particular stress combinations could persist during environmental periods that are less favorable for parental taxa.

### 4.2 Generation and strain specific responses among hybrid offspring

This study further showed that the responses of hybrid offspring to combined stress were determined not only by interspecific hybridization itself, but also by life-history generation and strain-specific genetic background (Ichihara et al., 2019). As summarized by Wichard and colleagues, *Ulva* species have an isomorphic alternation of generations, in which diploid sporophytes and haploid gametophytes are morphologically similar but differ in ploidy and reproductive origin (Wichard et al., 2015). This life-history feature provides a biological basis for expecting different physiological responses between F_1_ sporophytes and meiotically derived F_1_-derived gametophytes. In the present study, the F_1_ hybrid sporophyte was a diploid individual formed by the fusion of parental gametes and therefore theoretically carried genetic components from both *U. prolifera* and *U. linza*. In contrast, F_1_-derived gametophytes were haploid progeny derived from meiosis of the F_1_ sporophyte, and their genomes had undergone recombination and segregation. Therefore, differences in growth and photosynthetic pigment responses between F_1_ sporophytes and F_1_-derived gametophytes under combined stress are consistent with their distinct life-history stages and genetic backgrounds.

Across the two combined-stress experiments, the F_1_ hybrid sporophyte generally showed relatively stable photosynthetic pigment maintenance. This pattern was particularly evident in the temperature × desiccation experiment, where UP×UL1 generally maintained comparatively high Chl a, Chl b, and carotenoid contents across different temperature and water treatments. This suggests that the diploid hybrid sporophyte may possess a certain degree of physiological buffering, which may reflect enhanced physiological stability. Such stability may be associated with the coexistence of alleles from both parental species within the same genomic background, allowing more stable photosynthetic performance under environmental fluctuation. In contrast, the F_1_-derived gametophyte lines showed more divergent responses among treatments. Under some combined high-temperature–salinity and temperature–desiccation conditions, certain F_1_-derived gametophyte lines maintained higher growth rates or biomass accumulation, whereas others performed less well. Meiotic recombination and segregation can produce substantial variation among haploid progeny by reshuffling parental genomic components. Therefore, the divergent responses of UP×UL2 and UP×UL3 may reflect different combinations of parental alleles generated after meiosis (Stapley et al., 2017; Johnston, 2024).

Notably, differences among hybrid generations or strains were not always expressed through the same traits (Schnable and Springer, 2013). Some hybrid lines mainly showed higher growth rates or biomass accumulation, whereas others exhibited stronger photosynthetic pigment maintenance. This incomplete synchrony between growth and pigment traits suggests that different hybrid progeny may adopt different stress-response strategies. Some strains may prioritize rapid growth maintenance, while others may preferentially maintain photosynthetic structural stability or photoprotective capacity. Therefore, variation among F_1_-derived gametophyte lines may not simply reflect differences in overall vigor, but may also indicate physiological strategy differentiation resulting from distinct genetic combinations after meiosis.

### 4.3 Ecological implications for *Ulva* adaptation and green tide dynamics

The present findings have implications for understanding the adaptive evolution and potential ecological risks of green tide-associated *Ulva* taxa. *U. prolifera and U. linza* belong to a group of closely related *Ulva* lineages, and they show a certain degree of spatial and temporal overlap in coastal habitats and green tide source regions in China (Cui et al., 2015). Under some environmental conditions, they may also enter reproductive stages during overlapping periods (Liu et al., 2022). Therefore, if interspecific genetic exchange occurs in natural environments, the resulting ecophysiological variation in hybrid progeny may represent an important source of local adaptive potential. Under controlled experimental conditions, this study showed that hybrid offspring could maintain higher growth rates, biomass accumulation, or photosynthetic pigment levels under some combined-stress treatments, suggesting that interspecific hybridization may increase phenotypic and physiological diversity within *Ulva* populations across complex environmental gradients.

Coastal, intertidal, and green tide source habitats are characterized by strong environmental fluctuations, where elevated temperature, salinity variation, tidal emersion, and re-immersion often occur simultaneously rather than as isolated factors (Raven and Geider, 1988; Davison and Pearson, 1996; Kumar et al., 2014). Under such conditions, hybrid offspring with condition-dependent advantages may gain short-term growth or survival benefits in specific microhabitats (Arnold and Martin, 2010; Wang et al., 2024). For example, under combined high-temperature and high-salinity stress, low-temperature immersion, or high-temperature desiccation, some hybrid sporophytes or gametophyte lines showed stronger growth maintenance or pigment stability. These traits may help them maintain physiological activity in highly fluctuating coastal environments. Although such advantages do not necessarily translate directly into field dominance, they may provide additional phenotypic variation upon which environmental selection can act.

In the context of the Yellow Sea green tide, *U. prolifera* is regarded as the dominant causative species (Zhao et al., 2011), whereas *U. linza* often occurs as an important component of *Ulva* communities during early green tide development or in source-region habitats. If natural hybridization or localized gene flow occurs between these species, hybrid offspring may not necessarily become the dominant green tide biomass directly. However, novel genetic combinations and physiological variation generated by hybridization may influence the genetic structure and environmental responsiveness of green tide-associated *Ulva* assemblages (Payseur and Rieseberg, 2016). In particular, under global climate change and increasing coastal environmental variability (Capotondi et al., 2024; Krishna et al., 2025), hybrid genotypes with stronger tolerance to combined stress may be selectively favored within specific temporal or spatial windows, thereby potentially altering local population composition or increasing the adaptive potential of green tide-forming taxa.

It should be emphasized that this study was based mainly on growth and photosynthetic pigment responses under controlled laboratory conditions, and these results cannot directly demonstrate that hybrid offspring have competitive superiority or green tide-forming capacity in the field. Fitness in natural environments is also shaped by reproductive success, attachment ability, floating capacity, nutrient utilization, microbial interactions, grazing pressure, and hydrodynamic processes (Smetacek and Zingone, 2013). Therefore, the ecological significance of this study should be interpreted as evidence that interspecific hybridization may provide a source of novel physiological variation in *Ulva* populations, and such variation may have potential ecological consequences under complex environmental selection. Future studies integrating field-based population genetic surveys, long-term competition experiments, and multi-omics analyses will be necessary to further evaluate the actual role of natural hybridization between *U. prolifera* and *U. linza* in green tide formation and succession.

## 5 Conclusion

In conclusion, this study demonstrated that interspecific hybridization between *U. prolifera* and *U. linza* can generate condition-dependent variation in growth and photosynthetic pigment responses under combined temperature–salinity and temperature–desiccation stress. Hybrid offspring did not show a universal advantage over the parental strains, but some hybrid sporophytes and gametophyte lines maintained relatively higher growth, biomass accumulation, or pigment contents under specific stress combinations. The contrasting responses between the F_1_ hybrid sporophyte and meiotically derived F_1_ gametophyte lines further indicate that hybrid performance is both generation- and strain-specific. These findings suggest that interspecific hybridization may broaden physiological variation and adaptive potential in *Ulva* populations under fluctuating coastal environments. However, the ecological consequences of such hybrid variation in natural green tide systems require further validation through field surveys, long-term competition experiments, and molecular analyses.

## Data Availability

The raw data supporting the conclusions of this article will be made available by the authors, without undue reservation.
